# Factor structure of the General Health Questionnaire (GHQ-12) in subjects who had suffered from the 2004 Niigata-Chuetsu Earthquake in Japan: a community-based study

**DOI:** 10.1186/1471-2458-7-175

**Published:** 2007-07-24

**Authors:** Shin-ichi Toyabe, Toshiki Shioiri, Kuriko Kobayashi, Hideki Kuwabara, Masataka Koizumi, Taro Endo, Miki Ito, Hiroko Honma, Noboru Fukushima, Toshiyuki Someya, Kouhei Akazawa

**Affiliations:** 1 Department of Medical Informatics, Niigata University Medical and Dental Hospital, Asahimachi-Dori 1, Niigata 951-8520, Japan; 2 Department of Psychiatry, Niigata University Graduate School of Medical and Dental Sciences, Asahimachi-Dori 1, Niigata 951-8510, Japan; 3 Group of Mental Health, Section of Safety Net of Life, Research Center for Natural Hazards and Disaster Recovery, Niigata University, Igarashi-2-8050, Niigata 950-2181, Japan; 4 Niigata Institute for Traumatic Stress, Kawagishicho 1-57-1, Niigata 951, Japan; 5 Niigata Prefectural Mental Health and Welfare Center, Kamitokoro-2-2-3, Niigata 950-0994, Japan

## Abstract

**Background:**

Factor structure of the 12-item General Health Questionnaire (GHQ-12) was studied by a survey of subjects who had experienced the 2004 Niigata-Chuetsu earthquake (6.8 on the Richter scale) in Japan.

**Methods:**

Psychological distress was measured at two years after the earthquake by using GHQ-12 in 2,107 subjects (99.0% response rate) who suffered the earthquake. GHQ-12 was scored by binary, chronic and Likert scoring method. Confirmatory factor analysis was used to reveal the factor structure of GHQ-12. Categorical regression analysis was performed to evaluate the relationships between various background factors and GHQ-12 scores.

**Results:**

Confirmatory factor analysis revealed that the model consisting of the two factors and using chronic method gave the best goodness-of-fit among the various models for factor structure. Recovery in the scale for the factor 'social dysfunction' was remarkably impaired compared with that of the factor 'dysphoria'. Categorical regression analysis revealed that various factors, including advanced age, were associated with psychological distress. Advanced age affected the impaired recovery of factor 'social dysfunction' score as well as total GHQ score.

**Conclusion:**

The two-factor structure of GHQ-12 was conserved between the survey at five month and that at two years after the earthquake. Impaired recovery in the ability to cope with daily problems in the subjects who had experienced the earthquake was remarkable even at two years after the earthquake.

## Background

The twelve-item version of the General Health Questionnaire (GHQ-12) is used as a screening instrument for psychological distress in the general population [1,2]. GHQ-12 has been widely used as a unitary measure [3,4], but two or more underlying factors have been identified in previous studies based on factor analyses [5-7]. Scoring methods, clinical groups, different cultures and sampling time affected the number of factors that have been identified and the item loadings for each factor [5]. There have been many publications related to the psychological distress on survivors in a large disaster. They include devastating earthquake [8-10], air disaster [11], Chernobyl disaster [12], Severe Acute Respiratory Syndrome (SARS) epidemic [13] and war [14,15]. We previously reported psychological distress of subjects who had experienced the Niigata-Chuetsu Earthquake determined by using GHQ-12 at five months after the earthquake [16]. The earthquake (6.8 on the Richter scale) occurred at 5:56 P.M. on October 23, 2004 in the Niigata-Chuetsu region of Japan, and numerous aftershocks occurred until 28 December. More than 4,500 people were injured and 120,000 houses were completely or partially destroyed by the earthquake. Even at five months after the earthquake, 9,600 people who had lost their houses were living in temporary housing. At that time, recovery from the psychological distress caused by the earthquake was significantly impaired. A two-factor model using chronic scoring method [17] was found to show the highest level of goodness-of-fit, and the factor 'social dysfunction' was more severely affected than the factor 'dysphoria'. The impairment in the factor 'social dysfunction' was the most remarkable in the elderly and seemed to be a cause for the impaired psychological recovery in the elderly.

At two years after the earthquake, 4,500 people who had experienced the earthquake were still living in temporary housing. In those circumstances, we surveyed psychological distress of subjects using GHQ-12 again and analyzed the factor structure of the score. Our results showed that the two-factor structure was conserved over time and that impaired recovery of the factor 'social dysfunction' was apparent even two years after the earthquake.

## Methods

Two years after the Niigata-Chuetsu Earthquake, subjects who experienced the earthquake were asked to reply to questionnaires prepared to measure the level of their psychological distress. They lived in the area both when the earthquake occurred and when this survey was carried out. They were recruited by random sampling stratified by geographic areas affected by the earthquake. Psychological distress was measured by using the Japanese version of the 12-item General Health Questionnaire (GHQ-12) [1]. The GHQ-12 was scored by the binary [2], chronic and Likert scoring [18]. The proportions of subjects who suffering psychological distress were estimated by using cutoff points for GHQ scores. We used average GHQ scores as the cutoff points as recommended [19].

We performed confirmatory factor analysis in order to find the most fitted model. Confirmatory factor analysis was conducted using AMOS 7 (SPSS Japan Inc., Tokyo, Japan) to test the fits of various models [20]. The candidate models were constructed on the basis of models used in previous studies and those that we found by exploratory factor analysis [5,7,16,21-25]. Goodness-of-fit of the models was tested by using normal fit index (NFI), Tucker-Lewis coefficient (TLI), comparative fit index (CFI), root mean square error of approximation (RMSEA), Akaike information index (AIC), estimated population discrepancy (F0) and expected cross validation index (ECVI) [3,4,26]. Internal consistency of series of items belonging to each factor was evaluated using Cronbach's alpha score [27]. If Cronbach's alpha score of a factor was more than 0.7, we considered internal consistency of the factor to be satisfactory. In that case, we calculated lower scale points for each factor by averaging scales of all items belonging to the factor.

Categorical regression analysis was conducted to evaluate the impact of the subjects' background on GHQ scores. Categorical regression analysis quantifies categorical data by assigning numerical values to the categories, resulting in an optimal linear regression equation for the transformed variables [28-30]. We used the CATREG procedure of SPSS 15.0J (SPSS). Analysis of variance (ANOVA) with Scheffe post hoc tests and Jonckheere-Terpstra test were used to evaluate differences in GHQ scores between items specified by the categorical regression analysis. In all tests, a p-value less than 0.05 was considered to be statistically significant. Analyses other than confirmatory factor analysis were performed using SPSS 15.0J.

This study was approved by the Ethics Committee of Niigata Graduate School of Medical and Dental Sciences. Informed written consent was obtained from all subjects.

## Results

**Figure 1:**
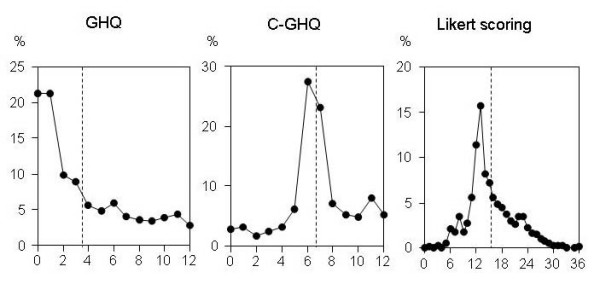
**Relative frequency polygons for GHQ-12 score**. GHQ-12 was scored by three different methods. In each method of scoring, the proportions of subjects with each score were plotted. Vertical dotted lines indicated the cutoff points for each scoring method.

Data were collected from 2,129 subjects, and 2,107 (99.0%) of the subjects responded to the questionnaire (Table 1). GHQ-12 scores were assessed by three different methods. The average GHQ-12 score were 3.6 ± 3.6 in binary method, 6.8 ± 2.7 in chronic method and 15.3 ± 5.3 in Likert method. When the average GHQ scores were used as cutoff points [19], the proportions of subjects who were considered to be suffering psychological distress were 33.1% in binary method, 53.3% in chronic method and 39.3% in Likert method (Fig. 1).

**Table 1:** Background of study subjects

item	category	number	%
Gender	Male	1,310	62.2
	Female	792	37.6
Age when the earthquake occurred (years)			
	-29	34	1.6
	30–39	184	8.7
	40–49	304	14.4
	50–64	805	38.2
	65–79	693	32.9
	80-	87	4.1
Place of residence when the earthquake occurred			
	Nagaoka City	794	37.7
	Ojiya City	395	18.7
	Mitusuke City	249	11.8
	Tohkamachi City	334	15.9
	Kawaguchi Town	85	4.0
	Koshiji Town	115	5.5
	Yamakoshi Village	115	5.5
Employment			
	Farmer	256	12.1
	Other self-employed individuals	238	11.3
	Office worker	643	30.5
	Part-time worker	143	6.8
	Housewife	278	13.2
	Student	1	0.0
	None	462	21.9
Total		2,107	

Confirmatory factor analyses were performed for various models, including the model that we found by exploratory factor analysis [5,7,16]. Among the various models, the two-factor model found by exploratory factor analysis showed more favorable fitting measures than those of the one-factor model and models consisting of three or more factors. Some of the results are presented in Table 2. In addition, the two-factor model using chronic method showed fitting measures superior to those of binary method and Likert method (Table 2), although there were no statistical differences in the fitting measures between the three scoring methods. Therefore, we used chronic method in subsequent analyses. The internal consistency of each factor in all three scoring methods was satisfactory, because Cronbach's alpha score for two factors ranged from 0.87 to 0.90.

**Table 2:** Fit measures of GHQ-12 scores for the one-factor model and two-factor model. GHQ-12 scores were evaluated by three different methods. The best fit measures are indicated by bold letters.

	1-factor solution			2-factor solution		
		
	binary	chronic	Likert	binary	chronic	Likert
χ^2^	2415.659	5780.508	3415.499	514.597	**303.553**	478.497
χ^2^/df	44.734	107.046	63.250	11.967	**7.059**	11.128
NFI	0.803	0.567	0.744	0.958	**0.977**	0.964
TLI	0.720	0.377	0.634	0.940	**0.970**	0.949
CFI	0.806	0.569	0.747	0.961	**0.980**	0.967
RMSEA	0.144	0.224	0.172	0.072	**0.054**	0.069
AIC	2487.659	5852.508	3487.499	582.597	**371.553**	546.497
F0	1.121	2.719	1.596	0.224	**0.124**	0.207
ECVI	1.181	2.779	1.656	0.277	**0.176**	0.259

**Figure 2:**
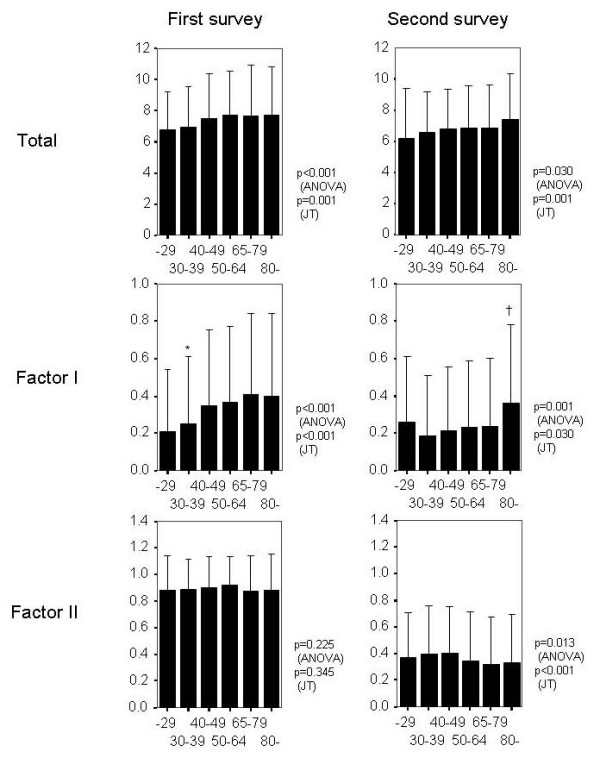
**Differences of lower scale points by age group of subjects**. The relationship between age of subjects and GHQ scores is shown as mean and standard deviation values. The data obtained two years after the earthquake (second survey) are shown with the data obtained five months after the earthquake (first survey). Trends of GHQ scores with increasing age of subjects were analyzed by ANOVA (ANOVA) with Scheffe post hoc tests and Jonckheere-Terpstra Test (JT). The p value of each test is shown in the plot. The factor I score of age group years 40–49 was significantly lower than groups years 65–79 and 80-(*) in the first survey. Age group years 80-showed significantly higher factor I score than other age groups (†).

To reveal what background factors were associated with GHQ-12 scores, we performed categorical regression analysis using background factors as independent factors (Table 3). Various factors were found to be associated with GHQ-12 scores. Among them, age of subjects was associated with total GHQ scores as well as the lower scale points for factor I. ANOVA revealed that there were significant differences in total GHQ scores, lower scale points for factor I and those for factor II between the age groups (Fig. 2). Age affected the lower scale point of each factor in a different manner. The lower scale points for factor I tended to increase with increase in age of subjects, whereas those for factor II tended to decrease.

**Table 3:** Factors that affect GHQ-12 scores. Results of categorical regression analysis are shown. In each analysis, dependent variables were total chronic score, lower scale point for factor I and that for factor II. Only factors with significant regression coefficients (p < 0.05) are shown.

	beta		
	
	Scoring method		
Items	total	Factor I	Factor II
Female gender	0.104	0.118	0.103
Age	0.047	0.061	-0.101
Married state	-	-	0.044
Kind of places of residence when the earthquake occurred	0.063	0.069	0.055
Kind of employment when the earthquake occurred	0.104	0.106	-0.155
No family member living together when the earthquake occurred	-	-	-
Accompanying person when the earthquake occurred	-	-	-
Severity of house damage caused by the earthquake	0.110	0.081	0.098
Kind of places of residence after the earthquake	-0.064	-0.057	0.043
Severity of injuries caused by the earthquake	-	-	0.059
Severity of sickness after the earthquake	0.146	0.160	0.146
Places living now	0.132	0.160	0.127
Employment now	0.156	0.137	0.120
No family member living together now	-	-	-
No changes in family members after the earthquake	-0.095	-0.100	-0.088
Consultation with persons after the earthquake	-0.102	-0.092	-0.100
Consultation with persons now	-0.070	-0.053	-0.108
			
Adjusted R-square	0.134	0.155	0.139

We compared data obtained two years after the earthquake (second survey) with data obtained five months after the earthquake (first survey) [16]. The average chronic score was significantly decreased at the second survey compared with that at the first survey (p < 0.001), and the decrease was quite remarkable in the factor II points (Fig. 2). On the other hand, the factor I points were less affected by time after the earthquake. The sustained high points of factor I were remarkable in subjects older than 29 years, especially in subjects more than 80 years old.

## Discussion

In the present study, we found that a considerable proportion of subjects who had experienced the Niigata-Chuetsu Earthquake had psychological distress even two years after the earthquake. The psychological distress assessed by GHQ-12 had a two-factor structure, and the factor 'social dysfunction' related to ability to cope with daily problems was more affected than the factor 'dysphoria'. Although various backgrounds of subjects were associated with impaired recovery from psychological distress, advanced age was associated with impaired recovery in the same manner as that in the survey five months after the earthquake. Aging affects psychological morbidity mainly through the factor 'social dysfunction', not through the factor 'dysphoria'.

We previously reported that a model consisting of the two factors showed a high level of goodness-of-fit in a survey of subjects who had experienced the Niigata-Chuetsu Earthquake at five months after the earthquake [16]. The proposed two-factor model also showed a good fit to the results of the survey of subjects two years after the earthquake. There has been debate as to whether GHQ is a uni-dimensional or multi-dimensional measure [3-6]. Our results regarding model fitting showed that the proposed two-factor model was superior to the one-factor model and models consisting of three or more factors (data not shown) [5,7]. The two-factor structure in the present study was quite stable regardless of the differences in scoring methods and sampling time. In general, the factor structure of GHQ-12 has provided quite different results in terms of scoring methods, clinical groups and different cultures [5]. However, the structure of the proposed model was very similar to that of a model developed by Doi, who assessed the factor structure of GHQ-12 in the Japanese general adult population [1]. The similarity in structure of models in their study and our study suggests that this model might be suitable for a survey in the Japanese population.

Among the two-factor models using three different scoring methods, the model constructed using chronic method showed the best fit. In chronic method, the response "no more than usual" to negatively worded questions in the questionnaire is scored 1 instead of 0 in the binary method. Goodchild et al. reported that such a response might represent an admission of a chronic problem rather than lack of a problem [17]. They suggested that the revised scoring method improves the problem of the response of the same subjects progressively diminishing during repeated surveys using binary method [31]. Although there is still debate as to whether chronic method really improves the sensitivity of GHQ [32-35], our results suggest that chronic scoring was the most suitable method for analyzing psychological distress that remained two years after the earthquake.

It is not known how long psychological distress remains in subjects who have suffered a devastating earthquake, especially when the psychological distress is evaluated by GHQ scores. Cao et al. reported that the proportion of subjects with psychological distress was higher than non-exposed controls at five months after the 1988 Yun Nan Earthquake using the Chinese version of GHQ-28 [10]. Carr et al. studied the time course of psychological distress over a period of two years after the 1989 Newcastle Earthquake, and they found that GHQ-12 scores sharply decreased during 12 months after the earthquake but tended to gradually decline further over time [8]. Therefore, it is not known when the suffering subjects would psychologically recover to the levels of control subjects in terms of GHQ-12 scores.

There are several limitations in this study. First, there were no non-exposed or pre-earthquake control subjects in this study. Second, no correspondence between the subjects in the first survey and those in the second survey exists. The backgrounds of the subjects in the two surveys were different, and the difference was especially remarkable in the gender ratio of study subjects. The male-to-female ratio was 55%:45% in the first survey, but it was 62%:38% in the second survey. Therefore, we did not directly compare the results of the two surveys but we used the results of first survey just for reference to the second survey. Nevertheless, it is obvious that the subjects were affected by matters related to the factor 'social dysfunction' two years after the earthquake and that subjects more than 80 years old were more affected than younger subjects.

## Conclusion

The two-factor structure of GHQ-12 was conserved between the survey at five month and that at two years after the earthquake. Impaired recovery in the ability to cope with daily problems in the subjects who had experienced the earthquake was remarkable even at two years after the earthquake.

## Competing interests

The author(s) declare that they have no competing interests.

## Authors' contributions

ST contributed to concept and design of the article and analysis of data. TS1, TS2 and KA supervised all aspects of the study. HK, MK, TE, MI, HH and NF contributed to acquisition of data. KK contributed to statistical analysis of data. All authors read and approved the final manuscript.

## Pre-publication history

The pre-publication history for this paper can be accessed here:


